# Recent advances in cochlear hair cell nanophysiology: subcellular compartmentalization of electrical signaling in compact sensory cells

**DOI:** 10.12703/r/9-24

**Published:** 2020-12-21

**Authors:** Thomas Effertz, Tobias Moser, Dominik Oliver

**Affiliations:** 1 InnerEarLab, Department of Otorhinolaryngology, University Medical Center Göttingen, 37099 Göttingen, Germany; 2 Institute for Auditory Neuroscience and InnerEarLab, University Medical Center Göttingen, 37099 Göttingen, Germany; 3 Auditory Neuroscience Group, Max Planck Institute for Experimental Medicine, 37075 Göttingen, Germany; 4 Synaptic Nanophysiology Group, Max Planck Institute for Biophysical Chemistry, 37077 Göttingen, Germany; 5 Multiscale Bioimaging Cluster of Excellence (MBExC), University of Göttingen, 37075 Göttingen, Germany; 6 Institute for Physiology and Pathophysiology, Philipps University, Deutschhausstraße 2, 35037 Marburg, Germany; 7 Center for Mind, Brain and Behavior (CMBB), Universities of Marburg and Giessen, Germany; 8 DFG Research Training Group, Membrane Plasticity in Tissue Development and Remodelling, GRK 2213, Philipps University, Marburg, Germany.

**Keywords:** Auditory hair cell, Mechanoelectrical transduction, Ion channel clustering, Synaptic physiology, Nanodomain, Phosphoinositide, Potassium channel, Compartmentalization, Basolateral membrane, Prestin, TMC1, Cav1.3

## Abstract

In recent years, genetics, physiology, and structural biology have advanced into the molecular details of the sensory physiology of auditory hair cells. Inner hair cells (IHCs) and outer hair cells (OHCs) mediate two key functions: active amplification and non-linear compression of cochlear vibrations by OHCs and sound encoding by IHCs at their afferent synapses with the spiral ganglion neurons. OHCs and IHCs share some molecular physiology, e.g. mechanotransduction at the apical hair bundles, ribbon-type presynaptic active zones, and ionic conductances in the basolateral membrane. Unique features enabling their specific function include prestin-based electromotility of OHCs and indefatigable transmitter release at the highest known rates by ribbon-type IHC active zones. Despite their compact morphology, the molecular machineries that either generate electrical signals or are driven by these signals are essentially all segregated into local subcellular structures. This review provides a brief account on recent insights into the molecular physiology of cochlear hair cells with a specific focus on organization into membrane domains.

## Introduction

The organ of Corti inside the cochlea forms the sensory organ of our sense of hearing. It houses two types of mechanosensory hair cells: three rows of outer hair cells (OHCs, approximately 12,000 in the human cochlea) and one row of inner hair cells (IHCs, approximately 3,500) span the entire length of the basilar membrane. Sound-induced vibrations of the tympanic membrane from approximately one Å are sufficient to elicit hair cell responses while vibrations of hundreds of nm are required to saturate that response. The ensuing receptor potential (RP) drives their key output function: active micromechanics (OHCs) and synaptic sound encoding (IHCs). OHCs and IHCs are highly specialized for operating at the fast pace inherent to auditory signaling. This applies to both the unique motor process of OHCs and the temporal bandwidth of signal transmission at the specialized ribbon synapses in IHCs. A sophisticated set of ion channels enables operation of these processes in the kHz range (OHCs) and hundreds of Hz range (IHCs). Both show afferent and efferent innervation but to different extents and serving different functions. Efferent control by medial olivocochlear projections is more prominent at OHCs, where an unconventional cholinergic mechanism inhibits electromotility (eM). Afferent synaptic transmission of IHCs to myelinated type I spiral ganglion neurons (SGNs; type I forming 95% of all SGNs) mediates cochlear sound encoding, while OHCs do not seem to contribute to sound encoding but instead signal to unmyelinated type II SGNs, likely to report cochlear damage. Once dysfunctional or lost, OHCs and IHCs cannot be replaced and sensory hearing loss results.

Mammalian hair cells are small and compact cells and therefore are also understood to be electrically compact, i.e. isoelectric. Nevertheless, recently, it has been demonstrated that IHCs of the rodent cochlea can undergo macromolecular and low-resistance electrical coupling to form functional mini-syncytia^1^, indications for which had previously also been reported for hair cells of the avian auditory papilla^2^. OHCs do not seem to engage in such direct coupling^1^ but interact mechanically^3^. More generally, neighboring hair cells face a common mechanical input resulting from the sound-driven traveling wave. At a subcellular level, it has become increasingly clear that essentially all of the various membrane specializations that generate and shape their electrical signals (i.e. RPs) on the one hand and the effectors that drive the cell’s output in response to the RP on the other hand are assembled into spatially confined membrane nanodomains rather than evenly distributed across the isoelectric membrane.

The higher-level principle governing the subcellular organization of hair cells is their nature as epithelial cells, imposing structural and functional polarization of the cell surface into apical and basolateral membranes. In all hair cells, the mechanoelectrical transduction (MET) machinery that provides the stimulus-modulated transducer conductance is found at the apical pole. Opposing the depolarizing apical MET conductance, basolateral potassium conductances establish the hair cell’s negative resting potential, whereby the balance between apical and basolateral conductances defines its actual value estimated as –40 and –55 mV in OHCs and IHCs, respectively^4^. Equally important, basolateral channels, including low-voltage activated Ca2^+^ channels, are essential for shaping the RP in terms of both amplitude dynamics and timing.

Finally, the effectors of the RP are also built into the basolateral membrane region. This is where functional divergence between IHCs and OHCs is fundamental: in the basolateral membrane of the IHCs, ribbon-type active zones (AZs) transmit temporal and amplitude information contained in the RP with unprecedented precision to the postsynaptic auditory neurons. In OHCs, in contrast, the RP primarily drives ultrafast cell length changes, termed eM, by a machinery that occupies large parts of the basolateral membrane domain.

In recent years, the nanoscale organization of several functional modules and their molecular constituents (and their interactions) has been resolved. While in some cases the physiological meaning of compartmentalization is well understood, e.g. to provide nanoscale coupling by the diffusible messenger Ca^2+^ or to avoid cross-talk of intracellular signals, in other cases the underlying subcellular physiology remains unknown or speculative. Here we attempt to describe recent progress in understanding the subcellular compartmentalization of membrane physiology in cochlear hair cells, the molecular composition of identified domains, and their functional relevance.

## The apical membrane: mechanoelectrical transduction

### Molecular composition of the MET channel complex

**Figure 1.**
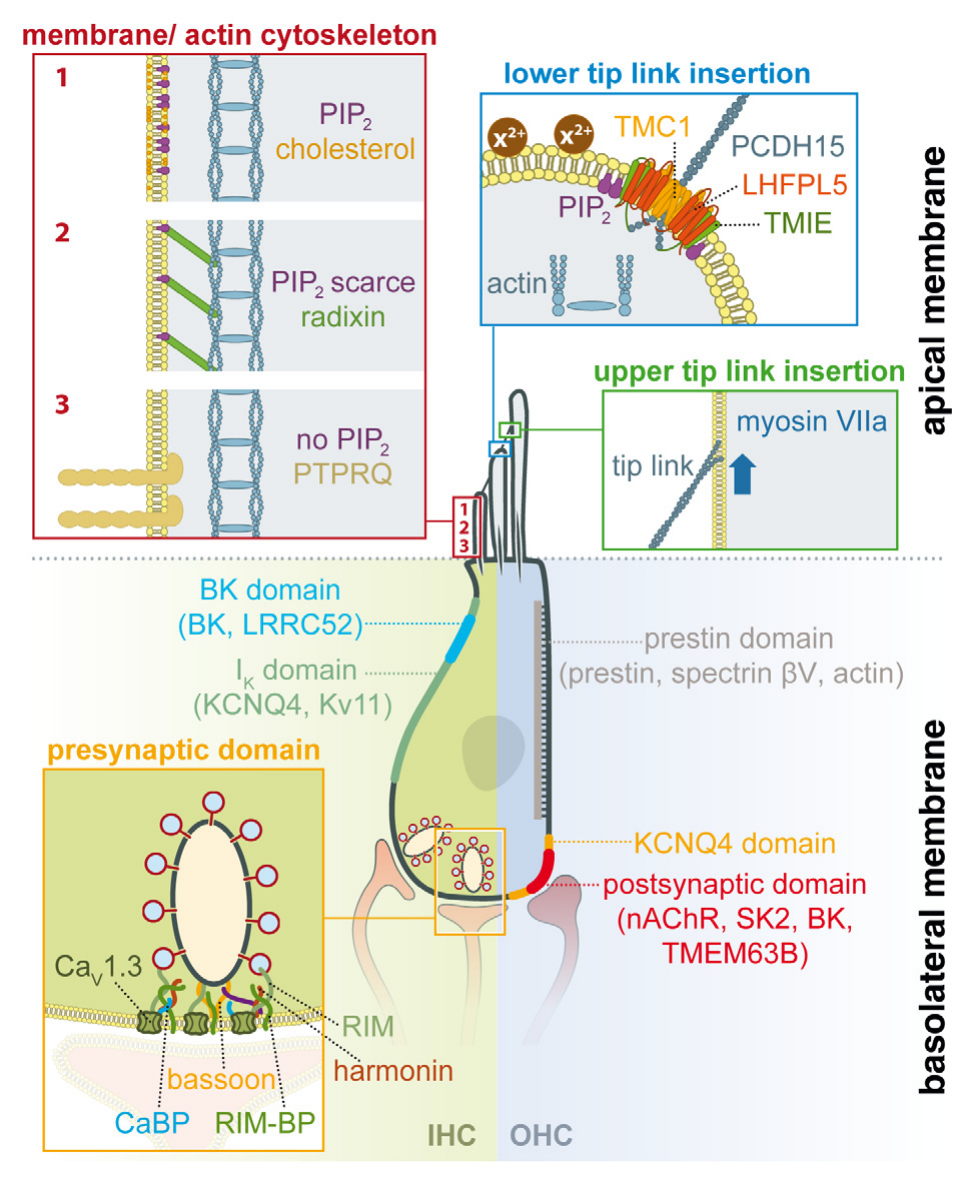
Domain organization of functional modules in the plasma membrane of inner hair cells (IHCs) and outer hair cells (OHCs). **Top section**: At the apical pole, the function and structural organization of mechanoelectrical transduction (MET) is similar for both hair cell types. Enlarged insets show simplified representations of stereociliary domains as defined by protein and lipid content as well as by function. Two major protein complexes define highly specialized domains that mediate MET and are located at the upper (green box) and lower (blue box) tip link insertion points (note that only a selection of proteins are shown for space reasons). At the lower tip link insertion point, the lower end of the tip link (protocadherin 15 [PCDH15] dimer) makes contact with the MET channel complex, where transmembrane channel-like protein isoform-1 (TMC1) and transmembrane inner ear (TMIE) are thought to constitute the MET channel pore and further components, including lipoma HMGIC fusion partner-like 5 (LHFPL5), phosphatidylinositol-4,5-bisphosphate (PIP_2_), and divalent cations, (X^2+^) affect pore properties and adaptation. At the upper tip link insertion point, myosin VIIa is now thought to maintain the tip link under tension rather than mediating fast adaptation, although modulation of myosin VIIa activity may affect the dynamic range of MET responses. Beyond these fundamental MET complexes, the stereociliary membrane (red box) is organized in distinct domains along the length of each stereocilium, which differ in protein and lipid content. Some identified components characterized so far are highlighted in the blow-up cartoons of three distinct regions (labeled 1–3) of each stereocilium. **Lower part**: The basolateral pole harbors distinct sets of functional modules in IHCs and OHCs, which are therefore shown separately. K^+^ ion channel populations are predominantly located towards the neck region in IHCs (left, light blue) but at a basal cap-like membrane area in OHCs (right, light yellow). Specifically, in OHCs, postsynaptic acetylcholine receptors (AChRs) and their downstream effectors, small conductance Ca^2+^-activated K^+^ channels, are clustered at the subsynaptic membrane of efferent cholinergic synapses. In both cell types, K^+^ channel positioning is reciprocal to the localization of the predominant effector modules. In the case of the IHC, this is the functionally heterogenous population of ribbon-type active zones each holding a Ca^2+^ channel cluster. The presynaptic scaffold bassoon, anchoring the ribbon and contributing to clustering CaV1.3 Ca^2+^ channels, is highlighted in the inset (orange box). The Ca^2+^ channel complex is composed primarily of the CaV1.3α, the CaVβ2, and CaVα2δ2 subunits and of interacting proteins such as Ca^2+^-binding proteins (CaBPs), harmonin, Rab3-interacting molecules (RIMs), and RIM-binding proteins (RIM-BPs). In the OHC, the entire lateral membrane is occupied by the machinery that generates electromotility (“prestin domain”), including densely packed prestin in the membrane and a poorly characterized actin-associated submembrane scaffold, lined by subsurface membrane compartments (cisternae). BK, Ca^2+^-activated potassium channel; LRRC, leucine-rich-repeat-containing; nAchR, nicotinic acetylcholine receptor; PTPRQ, protein tyrosine phosphatase receptor type Q; SK2, small conductance Ca^2+^-activated potassium channel; TMEM63B, transmembrane protein 63B.

A supramolecular MET complex is located at the tips of all but the tallest row of stereocilia, thus defining a highly specialized and spatially extremely confined domain (Figure 1) that also includes a specific membrane lipid composition (see below) and an interaction with a distinct lateral membrane domain in the neighboring, larger stereocilium, connected via the tip link.

Recent data show that the auditory MET complex involves intricate interactions among a set of proteins (i.e. transmembrane channel-like protein isoform-1/2 [TMC1/2]^5,6^, lipoma HMGIC fusion partner-like 5 [LHFPL5]^7,8^, transmembrane inner ear [TMIE]^9,10^, calcium‐ and integrin‐binding protein 2 [CIB2]^11^, and protocadherin 15 [PCDH15]^12^) and membrane lipids such as phosphatidylinositol-4,5-bisphosphate (PIP_2_)^10,13,14^ (Figure 1). Adding to the molecular intricacy of the MET complex is the variable temporal expression of its components reflected in an ongoing maturation process up until and after the onset of hearing, of which the TMC1/2 proteins are only one example^15–17^.

TMC1/2 proteins constitute a core component of the MET complex and are now thought to contribute to its pore-forming subunits^5,6,16,18–26^. While TMC2 is only transiently expressed in auditory hair cells during early postnatal development, TMC1 expression rises gradually and is essential for adult auditory MET^25^. Evidence for TMC proteins forming the MET pore comes, among others, from chemical modification of introduced cysteine residues, which altered ion selectivity of the MET currents^6,8,27^. Homology modeling of TMC structure with TMEM16 predicts two separate pores in a dimeric TMC complex, located at the margin of each subunit^24,25^. Interestingly, this model would see the pore “open” to the surrounding lipid bilayer laterally, rather than fully surrounded by protein. However, the TMC architecture might be complemented by TMIE, closing the pore towards the lipid bilayer^10^. Direct evidence that TMC1/2 proteins can form mechanosensitive ion channels was recently shown when orthologues of human TMC1/2 were successfully purified and reconstituted in liposomes and their mechanosensitivity demonstrated^26^. This study used CmTMC1 (green sea turtle, *Chelonia mydas*) and MuTMC2 (budgerigar, *Melopsittacus undulatus*), which possess 72% and 66% sequence identity to human TMCs, respectively. Verification of mechano-gating for mammalian TMC1 as well as further evidence in support of the current model of TMC1 dimers with two independent pores^25^ will require future work. Of note, while quasi-simultaneous^27,28^ opening of both pores seems possible, their simultaneous closure is harder to envision. A temporally linked but separated closing of two pores, consistent with stochastic single pore properties, has not been observed directly in previous single channel recordings^14,29,30^.

### MET current adaptation

Traditionally and primarily based on work in turtle and bullfrog hair cells, adaptation of the MET current was thought to be an exclusively Ca^2+^-driven process^31–33^ that modulates the MET channel’s resting open probability and thus its dynamic current/stimulus relationship^34–36^. Ca^2+^ was thought to interact either with the MET channel itself^32,37,38^ or act on myosin motors^33,39,40^ that slip or climb along the stereocilia actin core, thus controlling tip link tension. However, data showed that MET channels are present only at the tip of stereocilia and the presumed myosin “adaptation” motors at the upper tip link insertion point, making a modulation of those motors by Ca^2+^ entering through the MET channel unlikely.

After some controversial data on the matter^41–43^, recently, three different mechanisms controlling the MET channel resting open probability were proposed: i) a resting tension-generating, myosin-based process^44,45^ (Figure 1), ii) a fast, Ca^2+^-independent adaptation process^41^, and iii) an effect of extracellular Ca^2+^ that interacts with the lipid bilayer and modulates membrane stiffness^44^.

Reconciliation of Ca^2+^-dependent and Ca^2+^-independent models for adaptation came from comparison of the different stimulus methods used in previous studies. Where a fluid jet stimulates the hair bundle with a steady force (force clamp), a stiff probe stimulates the hair bundle with a constant displacement (displacement clamp). These two stimulation paradigms can either obscure or reveal Ca^2+^-dependent and -independent processes^46^. These data also suggested that the “natural” stimuli detected by IHCs and OHCs are different: the free-standing IHCs are driven by force (as with the fluid jet), while the OHCs are stimulated by displacement prescribed by the overlying tectorial membrane, which OHC hair bundles are connected to (as with the stiff probe)^42^.

### Lipid–MET channel complex interactions

In addition to the involved proteins and Ca^2+^ ions, the lipid bilayer, its organization, and its nanodomain properties are most certainly important for normal MET channel function. Close functional interaction of ion channel complexes with their lipid environment is a well-established concept^47–60^ (for review, see 61) and has recently become recognized more in auditory MET research^10,13,14,62,63^. For example, modulation of MET channel function by PIP_2_ was originally described in bullfrog hair cells^13^ and also demonstrated in murine IHCs^14^. Models on the role of the cell membrane for MET include the formation of cholesterol-rich force foci^64^, the relaying of force^44^, activation and/or modulation of MET channels^63^, or action as a functional cofactor of MET channel complexes^10,13,14,62,65^ (short review see 56).

### Distinct membrane domains along the mechanosensory stereocilium

While we are missing membrane composition data for IHCs and OHCs, some data derived from chick and bullfrog hair cells are available. These data show that even the membrane of each stereocilium shows distinct membrane domains along its height of just a few µm^66–68^ (Figure 1). Formation of membrane nanodomains containing distinct subsets of proteins and a close linkage between the actin core of stereocilia and the cell membrane were demonstrated for chick vestibular hair cells^66^. These data showed, for example, that cholesterol was enriched at the tips of stereocilia, where it potentially stiffens the membrane and could function as a force focus for the MET complex^61,64,69^. Other data also showed that PIP_2_ modulates core MET channel properties^13,14^, possibly due to direct interaction with TMIE^10^. Moreover, while PIP_2_ likely interacts with the MET complex, it is completely absent from the stereocilia ankle region, where PTPRQ, a putative lipid phosphatase, or radixin are prominently localized^66,67^. Likewise, the shaft region of each stereocilium shows a high concentration of the Ca^2+^ pump PMCA2^66^, which seems critical for Ca^2+^ clearance and in forming a distinct separation of cytosolic Ca^2+^ pools between stereocilia tips and ankles. Taken together, the data found in the non-auditory MET fields are so compelling^48,49,52,56,58,60,61^ that a close inspection of the dependence of the auditory MET complex on its cell membrane environment is warranted.

However, a detailed proteomic or lipidomic analysis of the stereociliary membrane in murine hair bundles is currently missing but necessary, as lipid domain differences between chick vestibular hair bundles and auditory murine hair bundles are known^14,66^. New methods, both nanoscopic and “omic”, will be required for investigating the morphology and dynamics of stereocilia nanodomains that are below the optical resolution limit of conventional light microscopes.

## The basolateral membrane

### Prestin-based electromotility

In the OHC, the RP drives eM, which is mediated by prestin (SLC26A5, Figure 1), a member of the SLC26 anion transporter family. Biophysical considerations and a wealth of experimental data indicate that voltage-dependent conformational rearrangements of prestin alter its dimension in the membrane, which at the population level translates into surface area changes of the membrane and hence cellular shape changes (area motor mechanism; for a comprehensive review, see 70). The predominant view for many years has been that this process is ultrafast, i.e. following acoustic frequencies far into the ultrasonic range (measurements ranging up to 80 kHz^71^). Such speed is the underlying assumption for many models of cochlear amplification, where eM amplifies cochlear vibration in a cycle-by-cycle manner. However, a series of recent detailed analyses of prestin and eM kinetics challenged this view, reporting lowpass behavior with corner frequencies at around 20 kHz, well below the upper frequency limit of hearing in many mammals, which obviously urges a reconsideration of the mechanism of cochlear amplification^72,73^.

Recent advances at the structural level are beginning to shed light on the molecular mechanisms underlying eM. Combined homology modeling, molecular dynamics simulations, and functional analysis clarified the membrane architecture and domain organization of the prestin protomer, showing a 7+7 inverted repeat architecture, and organization into two major opposing domains, tentatively termed gate and core domains^74^. This model was soon confirmed by the first experimental crystallographic structure of a bacterial SLC26 homolog^75^. Subsequent structural and biochemical analysis revealed that SLC26 proteins, including prestin, are dimers^76^. Finally, the first experimental structure of a eukaryotic homolog, mouse SLC26A9, recently uncovered additional details, particularly on substrate binding and unexpected involvement of the conserved intracellular C-terminus (STAS domain) in the dimerization of SLC26 proteins^77^. Together, all of these structural findings suggest that SLC26 transporters may act by an elevator mechanism, in which a mobile domain (core or transport domain) binds the substrate and delivers it to the other side of the membrane by a rotational–translational movement, with the other main domain (gate or scaffold domain) acting as a static scaffold. It will be exciting to see if the eM mechanism of prestin can also be understood in the framework of such an elevator mechanism.

Irrespective of the molecular details, the area motor mechanism requires large numbers of elementary motors in series to generate substantial macroscopic cellular motion or force. In fact, the entire lateral membrane consists of a dense array of particles believed to be individual prestin molecules^78^, whereas the basal area of the membrane is largely devoid of prestin (e.g. see 79), establishing a clear-cut segregation of the basolateral membrane into a lateral “prestin domain” and a basal subnuclear cap that essentially contains all other signaling functionality. This segregation goes along with low diffusional mobility of prestin in the lateral domain^80^.

Remarkably, in contrast to progress in understanding the molecular mechanisms of prestin, how the prestin domain is organized and held together has remained enigmatic. The OHC lateral wall is a multi-layered structure that, beyond the prestin-populated plasma membrane (Figure 1), also features a highly regular submembrane actin lattice and one or more subsurface cisternae that line up with most of the lateral membrane^78^. One identified component of this structure is the nonconventional spectrin βV^81^, whose localization to the lateral membrane parallels the evolutionary emergence of the lateral prestin domain^82^. Electron tomography revealed an even higher complexity and regularity of the entire trilaminate structure, including regularly spaced filamentous connectors between the actin lattice and both the plasma membrane and the cisternal membrane^83^. However, the identity of these components and their contribution to the formation and function of the prestin domain remain unknown. There is some evidence that prestin itself contributes to establishing lateral membrane identity in OHCs, as its genetic deletion leads to the relocation of other membrane proteins from their usual place into the lateral membrane^84^. A specific lipid composition may also contribute to the identity of the prestin domain. Thus, prestin’s diffusional mobility and function depend on cholesterol content^80,85^, and spectrin βV connects to the membrane via a phosphoinositide-specific PH domain^82^.

### Domain organization of basolateral ion channels

***OHCs*.** OHCs are characterized by a large basolateral K^+^ conductance that is key to ensuring a fast electrical membrane time constant permissive for alternating current RPs at acoustic frequencies^4^. Such fast electrical oscillation may be required to drive eM cycle-by-cycle (see above). The major OHC current is known as I_K,n_ for its unusually negative voltage range of activation, resulting in constitutive activity at physiological membrane voltages. The underlying K^+^ channels contain the pore-forming KCNQ4 subunit (Kv7.4)^86^. KCNQ4 proteins localize to the basal cap of the OHC^86^. This localization appears straightforward in avoiding interference with the high prestin density required in the lateral domain. Moreover, a recent report suggests that clustering of KCNQ4 may enforce cooperativity that alters functional properties^87^. In particular, clustering was shown to speed up gating kinetics and contribute to shifting the voltage range of activation, which in OHCs is much more negative than of heterologous KCNQ4 channels, a puzzle so far unexplained.

At least three additional ion channels are also clustered in the basal KCNQ4-positive domain (Figure 1): α9/10 nicotinic ACh receptors as well as SK2 and BK Ca^2+^-activated K^+^ channels. These three channel types constitute the postsynaptic ion channel complement of cholinergic olivocochlear inhibitory synapses and mediate unusual inhibitory postsynaptic potentials by an indirect ionotropic mechanism: the hair cell’s ionotropic α9/α10 cholinergic receptors are highly Ca^2+^-permeable channels^88^. Ca^2+^ influx rapidly activates colocalized Ca^2+^-activated K^+^ channels, resulting in net outward current and hence hyperpolarization. For a long time, it has been known that small-conductance SK2 channels mediate the outward current, and accordingly their localization is confined to the subsynaptic membrane, outlining this functional domain^89,90^ (Figure 1). However, large-conductance BK channels also contribute to the cholinergic hyperpolarization, particularly in the mid- to high-frequency regions of the cochlea. Indeed, BK and SK2 channels co-occupy the same synaptic membrane area^91,92^.

A recent unexpected addition to the ion channel complement at this domain is TMEM63B^93^. TMEM63B forms Ca^2+^-permeable cation channels activated by hypo-osmotic stress. It was shown that upon osmotic swelling of OHCs, TMEM63B channels are activated and initiate a regulatory volume decrease, presumably by activating cation efflux via Ca^2+^-activated K^+^ channels. Indeed, TMEM63B proteins are colocalized with SK2 channels in OHCs, suggesting that they, too, are part of the efferent postsynaptic membrane. The osmosensory function of TMEM63B appears to be important for OHC maintenance, since genetic deletion results in OHC degeneration and hearing loss^93^.

As for the prestin domain, the mechanisms and molecular interactions that target and restrict ion channels to their basal territory remain to be elucidated. Nevertheless, the spatial separation of basal channel domain and lateral prestin domain first emerges during postnatal development in rodents and is dependent on thyroid hormone signaling^79,90^, which might provide an experimental entry point towards the identification of molecular components and mechanisms involved in domain formation^94^.

***IHCs*.** IHCs express a surprisingly complex repertoire of basolateral potassium channels that has only recently been dissected in detail^95^. For decades, three major K^+^ conductances have been known, set apart by their distinct activation kinetics, voltage dependence, and pharmacology. The molecular identity of the channels that mediate two of these components (historically termed I_K,f_ for the fast activation kinetics and I_K,n_ for the particularly negative activation voltage range) is well understood. Large-conductance BK channels underlie I_K,f_^96,97^, and KCNQ4 (Kv7.4) channels mediate I_K,n_^98^. However, the channel(s) underlying the slowly activating K^+^ current I_K,s_ remained unknown. Dierich *et al*.^95^ combined expression data with pharmacology and biophysical characterization to dissect I_K,s_ and found that it is mediated by K_v_1.8, K_v_11.1, and K_v_12.1 channels. Modeling IHC RPs indicated that the complex K^+^ channel complement helps to ensure temporally precise signal encoding over a wide dynamic range^95^.

Under the assumption that the IHC is electrically compact, there is no obvious reason for spatial segregation of these channels, and an electrical point model was sufficient to produce all the properties of recorded RPs. Nevertheless, BK distribution is highly clustered into patches close to the neck region of IHCs^97 ^(Figure 1). This is particularly surprising since BK channels generally require elevated Ca^2+^ for activation within physiological voltage ranges^99^, whereas IHC Ca^2+^ channels are compartmentalized into the presynaptic zone of the IHC (see below), too far away to supply BK clusters with Ca^2+^ signals. Two papers now reveal a molecular mechanism that elegantly reconciles both issues: IHCs express LRRC52 as a BK-interacting protein^100,101^. LRRC52 acts as a gating modifier, strongly shifting the voltage sensitivity into a range that matches the hair cell’s physiological membrane potential even in the absence of elevated Ca^2+^^101^, a property previously determined for BK channels in IHCs^102^. Moreover, LRRC52 is responsible for clustering BK at the cell’s neck region, where basolateral Ca^2+^ signaling is out of reach^101^.

What about localization of the other K^+^ channel populations in the same cell? Lower-resolution immunohistochemistry is consistent with predominant localization of KCNQ4 to the upper part of the lateral membrane^98^. Similarly, Kv11.1 immunoreactivity is mainly found in the upper half of the lateral membrane^95^.

The physiological meaning of molecular sequestration of BK into distinct clusters and together with the other K^+^ channel entities into the upper region of the IHC remains elusive; a possible rationale may be to keep large K^+^ outward fluxes away from the synapses, where they could depolarize the postsynaptic endings, interfering with precise signal encoding^103^.

### Transmitter release by IHCs

During the last few years, substantial progress has been made toward the understanding of hair cell synaptic transmitter release, and much has been covered in elaborate reviews^104–109^. Hallmarks of the hair cell synapses are i) electron-dense synaptic ribbons, which provide AZ scaffolding, supply synaptic vesicles (SVs) and require the core component RIBEYE^110,111^ and the anchoring scaffold bassoon^112–115^, ii) exocytosis at high rates and with utmost temporal precision that is sustained over long periods of time by efficient endocytic SV recycling and SV replenishment to the large readily releasable pool of SVs^110,116–124^, and iii) heterogeneity of presynaptic structure and function that likely enables IHCs to decompose sound intensity information into different neural channels^125–129^.

Release occurs at presynaptic AZs, which are highly specialized nanodomains of the basolateral IHC membrane (Figure 1). It has become clear that the underpinning molecular composition deviates from that of conventional synapses and even from retinal ribbon synapses, likely to support the specific functional requirements of synaptic sound encoding (recent review in ^104,108^). For example, the Ca_V_1.3-based^130–132^ presynaptic Ca^2+^ channel complex is active already at the IHC resting potential at the subset of synapses that likely encode soft sounds and inactivates fairly little to reliable signal sound intensity. Beyond splicing of the pore-forming Ca_V_1.3α subunit^126,133^, choice of the auxiliary Ca_V_β and Ca_V_α2δ subunits^134–136^, interacting proteins such as Ca^2+^-binding proteins^137,138^, harmonin^126,139^, RIMs^121,140^, and RIM-BPs^120^ shape the precise biophysical properties of the channels at a given AZ (Figure 1).

Ca_V_1.3 channel complexes are clustered at a very high density of approximately 3,000 channels per µm^2^ at the large ribbon-type AZs (approximately 0.04 µm^2^)^127^ of transmitter release of the basolateral IHC membrane, while estimates of the extrasynaptic channel density are much lower (<10 channels per µm^2^ in mature IHCs)^141^. This compartmentalization of Ca^2+^ influx requires the action of multidomain proteins of the AZ: their deletion generally reduces the number of Ca^2+^ channels available for triggering SV release^114,120,121^ and can also alter the Ca^2+^ channel topography^120,127^. In the case of bassoon deletion, Ca^2+^ channels do not properly cluster^127^. Interestingly, even deletion of RIBEYE, not suspected to directly interact with Ca^2+^ channels, altered the topography and function of the Ca^2+^ channels^110^. The multidomain proteins of the AZ and the “superscaffolding” synaptic ribbon are likely to stabilize the Ca^2+^ channel cluster that faces massive membrane turnover due to exocytosis and endocytosis at the AZ and periactive zone membrane domains, respectively. So far, estimates of Ca^2+^ channel mobility have not been obtained for hair cell AZs, but studies in cultured neurons imply that it might be substantial^142^.

Single channel properties of Ca_V_1.3-based Ca^2+^ channels of hair cells have been assessed by cell-attached recordings^143–149^ as well as by non-stationary variance analysis^127,150,151^. There is consensus on the average total number of activatable Ca^2+^ channels per AZ (100) but likely method-related partial discrepancies for estimates of open probability and single channel current. Interestingly, evidence for cooperative gating, revealed for heterologously expressed Ca_V_1.3 channels^152^, has not been reported thus far. Recently, the presynaptic complement Ca^2+^ channel was revisited using Ca^2+^ imaging^126,127,153,154^, and a range of 20–300 channels per AZ was estimated^127^. Such massive differences in Ca^2+^ channel clustering are likely to impact their biophysical properties and the coupling of Ca^2+^ influx to SV release. Indeed, recently, it was shown that AZs vary in the control of exocytosis by Ca^2+^ influx^155^. However, the expectation that membrane nanodomains with a high density of Ca^2+^ channels might present an active local depolarization exceeding the overall hair cell potential seems not to be supported: the voltage dependence of activation does not correlate positively with the maximal Ca^2+^ influx^126^. A negative feedback to presynaptic Ca^2+^ influx is mediated by the release of protons during SV exocytosis that partially inhibit the Ca^2+^ channels under physiological pH buffering^156^, and a potential modulation of the IHC Ca^2+^ channel by lipids^157,158^ remains to be elucidated. Despite the heterogeneity of the control of exocytosis by Ca^2+^ influx among hair cell AZs, the voltage dependence of Ca^2+^ influx generally predicts that of SV release^155^. Site-specific trafficking of components of the Ca^2+^ channel complex might involve adapter proteins such as Gipc3^126^, and differences in the capacity of tethering multidomain proteins available at the AZ^114,120,121^ likely contribute to the heterogeneous Ca^2+^ channel complement. Recently, several candidate mechanisms for setting up AZ heterogeneity have been proposed: planar cell polarity signaling^159^, instruction by postsynaptic neurons^160^, and efferent olivocochlear signaling^161^. Finally, from the perspective of sound coding in the auditory system, it will be of utmost importance to elucidate how properties of the presynaptic AZs relate to the functional^162–166^ and molecular^128,129,160,167^ diversity of SGNs.

## Conclusion

In summary, the auditory hair cells provide an impressive model system for compartmentalization of electrical signaling in compact cells. Already the mechanosensory hair bundle shows different compartments and sub-domains which are essential for the reception and processing of mechanical stimuli. This is likely true for the largest (5 µm) and smallest (<500 nm) stereocilia. Although our technological means are insufficient, as of yet, to directly investigate the interplay of single lipid molecules or rafts with different proteins, pharmacological and genetic manipulations show a close interaction between the cell membrane and proteins, where alterations in either can cause deafness. Understanding these interactions not only at the tip region (MET-complex) but also along the shaft and in the ankles of stereocilia will be a crucial and painstaking endeavor.

For some functional units, particularly afferent and efferent synapses, co-localization of Ca^2+^ sources and Ca^2+^-driven effectors provide the obvious rationale for tight clustering of their components into precisely defined domains. Furthermore, this example also shows how the cellular domain architecture translates into functional separation of events even in a compact cell. Thus, in prehearing IHCs, transient efferent innervation results in interspersed afferent and efferent synapses, both driven by Ca^2+^ signals. Although the distances between both structures are in the low μm range, Ca^2+^ signals produced at efferent sites fail to elicit vesicular release at nearby afferent presynapses, demonstrating that the functional architecture associated with each domain and the efficient cellular Ca^2+^ handling ensure functional isolation^168^.

Moreover, detailed studies of the molecular components of the IHC ribbon synapse showcase how subcellular membrane domains are assembled. Nevertheless, it will be an important task for the future to elucidate how differential control of SV release by Ca^2+^ channels is established, potentially by varying the topography of release sites and Ca^2+^ channels or simply by domain overlap in the case of large Ca^2+^ channel clusters. The fascinating question of how hair cells establish subcellular heterogeneity among their Ca^2+^ channel clusters and, more generally, the membrane nanodomains of the AZs remains to be elucidated.

A similarly stringent analysis of the makeup of the other basolateral domains, in particular the OHC lateral membrane, is still outstanding and promises to reveal important insights into the micro- and nano-mechanics of electromotility.

Finally, in addition to the sophisticated assemblies of membrane proteins and membrane-associated proteins, local differences in lipid composition across the various domains are likely to contribute to their identity and functionality. Tools for tracking and manipulating individual lipid species in living cells have been developed in recent years^169,170^ and promise to provide insights into the role of lipids in hair cell function.
